# Variation in Gastric pH May Determine Kiwifruit’s Effect on Functional GI Disorder: An *in Vitro* Study

**DOI:** 10.3390/nu6041488

**Published:** 2014-04-11

**Authors:** Bruce Donaldson, Elaine Rush, Owen Young, Ray Winger

**Affiliations:** 1 Faculty of Health and Environmental Sciences, AUT University, Private Bag 92006, Auckland 1142, New Zealand; E-Mails: elaine.rush@aut.ac.nz (E.R.); owen.young@aut.ac.nz (O.Y.); 2 Institute of Food, Nutrition and Human Health, Albany Campus, Massey University, Auckland 0745, New Zealand; E-Mail: Ray.winger@btconnect.com

**Keywords:** kiwifruit, protease, gastric protein digestion, functional gastrointestinal disorder

## Abstract

Consumption of kiwifruit is reported to relieve symptoms of functional gastrointestinal (GI) disorder. The effect may be related to the proteases in kiwifruit. This *in vitro* study aimed to measure protein hydrolysis due to kiwifruit protease under gastric and duodenal conditions. A sequence of experiments incubated meat protein, with and without kiwifruit, with varying concentrations of pepsin and hydrochloric acid, at 37 °C for 60 min over the pH range 1.3–6.2 to simulate gastric digestion. Duodenal digestion was simulated by a further 120 min incubation at pH 6.4. Protein digestion efficiency was determined by comparing Kjeldahl nitrogen in pre- and post-digests. Where acid and pepsin concentrations were optimal for peptic digestion, hydrolysis was 80% effective and addition of kiwifruit made little difference. When pH was increased to 3.1 and pepsin activity reduced, hydrolysis decreased by 75%; addition of kiwifruit to this milieu more than doubled protein hydrolysis. This *in vitro* study has shown, when gastric pH is elevated, the addition of kiwifruit can double the rate of hydrolysis of meat protein. This novel finding supports the hypothesis that consumption of kiwifruit with a meal can increase the rate of protein hydrolysis, which may explain how kiwifruit relieves functional GI disorder.

## 1. Introduction

Clinical evidence [1] supports the hypothesis that for some people, consumption of kiwifruit (*Actinidia deliciosa*, Hayward variety) relieves symptoms of gastrointestinal (GI) disorder such as indigestion, bloating and constipation. Occurring more frequently with ageing [2], constipation, a symptom of functional GI disorder [3], has a multifactorial etiology including enforced immobility, autoimmune responses, microbial influx, inflammatory bowel disease, a change of diet or an unbalanced diet, and some forms of medication (including analgesics, antibiotics and chemotherapeutics) [4]. It is common practice in both New Zealand hospitals and elder care homes to administer Kiwifruit juice in the form of Kiwi Crush™ to relieve and prevent constipation. Treatment of constipation, among patients at Wellington Hospital with Kiwi Crush™, was reported as an effective option to reduce frequency of constipation and use of other laxative products’ [5]. The daily inclusion of fresh kiwifruit in the diet of the elderly has also been reported to increase the frequency and ease of laxation [6,7]. Furthermore, a hospital audit has shown the efficacy of inclusion of kiwifruit in the diet for the management of functional GI disorder [8].

An unusual feature of the mature green-fleshed Hayward variety kiwifruit that is used exclusively in the production of Kiwi Crush™, is the presence of protein (about 3 g/kg of fruit). Approximately 40% (≈1.2 g/kg of fruit) of the protein comprises a clade of six cysteine proteases, referred to collectively as actinidin [9]. The action of the proteases of kiwifruit under varying conditions of gastric digestion followed by duodenal digestion is not known.

The aim of this study was to measure the rate of protein hydrolysis with and without the kiwifruit protease by varying *in vitro* conditions that simulate gastric and duodenal digestion to test the hypothesis that consumption of kiwifruit with a meal can increase the rate and extent of the hydrolysis of protein; a measure of protein digestion efficiency.

## 2. Experimental Section

A sequence of timed, *in vitro*, protein-digestion experiments was undertaken. Components of the digestion milieu were meat protein, kiwifruit protease, pepsin, hydrochloric acid and water. Gastric digestion was at 37 °C for 60 min over the pH range 1.3–6.2 to simulate gastric digestion. Duodenal digestion was simulated by a further 120 min incubation at pH 6.4. Total ammoniacal nitrogen (N) in the components of the digestate was determined by Kjeldahl analysis pre-digestion. Details of components and the sequence followed are described below.

Each experimental run took almost six hours to complete and consisted of eight different treatments, plus a reference standard of meat protein in either water, 0.074 M HCl or 0.022 M HCl, with no added protease. The data from a run was discarded and repeated if the reference standard was more than ±0.5% of the expected value. Each experimental run was repeated the following day and if the ammoniacal N in any individual treatment did not vary from its replicate by more than 2%, the outcome was accepted, otherwise the run was repeated.

### 2.1. Protein and Protease

A homogenous freeze-dried, meat powder (FDM) was prepared from selected forequarter bovine shoulder steaks with a high component of connective tissue. All visible fat was trimmed and discarded and the meat double-passed through a 6-mm industrial mincing plate. The minced meat was boiled in water for 10 min and with the water, freeze-dried, milled to a powder and thoroughly mixed prior to packing into 50 g foil bags for refrigerated storage at −18 °C. The yield of FDM to wet weight of cooked meat pre drying was 30%, therefore 600 mg of FDM was equivalent to 2000 mg of hydrated cooked meat, the sample size recommended for Kjeldahl analysis [10].

Porcine pepsin (Sigma-Aldrich Australia, Product # P7125, with ≥400 Sigma units/mg protein, Sydney, Australia) was used to simulate human gastric pepsin. The other protease was the kiwifruit and we chose to use freeze-dried kiwifruit (Zyactinase^®^ from Vital Food Processors Ltd., Auckland, New Zealand). This purer form of kiwifruit enzyme (KF) was chosen because it provided a shelf-stable, homogeneous product (water content < 4%). The protease activity of KF, measured using a casein substrate, was ≥3000 units/mg of protein (manufacturer’s specification). 

The protein concentration of each ingredient (FDM, pepsin and KF) was determined from six replicate Kjeldahl analyses (see Section 2.3) of nitrogen, assuming a conversion factor of 6.25 g protein/g nitrogen. The mean concentrations of protein (g/100 g) and standard deviation for each were: FDM 85.25 ± 0.32; pepsin 60.20 ± 1.75; and for KF 4.34 ± 0.09 respectively. 

### 2.2. Primary and Post-Primary Digestions

The simple *in vitro* model digester used was comprised of 8 × 75 mL screw-top glass tubes held in a reciprocating flask shaker, set at 240 cycles per minute. The tubes were immersed in a 37 °C water bath. The sequence of experiments initially simulated ‘primary’ gastric digestion over a pH range 1.3 to 6.1 and explored the effect of protease concentrations ranging from 5 to 40 mg ± 1% for pepsin and 50 to 350 mg ± 1% for KF. Incubation-time effects on primary protein hydrolysis were also tested for KF over the range 15 to 120 min. The subsequent simulated “post-primary” duodenal digestion was fixed at pH 6.4 for 120 min. A 600 ± 0.5 mg sample of FDM and one or both proteases were weighed into each of eight glass tubes, a 20.0 mL aliquot of hydrochloric acid was added and the mixture incubated (37 °C) for 60 min for the primary digestion and an additional 120 min for the post-primary digestion. For the post-primary digestion pH was adjusted to 6.4 by adding phosphate buffered bicarbonate (0.074 M NaHCO_3_). After incubation, the mixtures were centrifuged at 2080 relative centrifugal force for 10 min and decanted into 250 mL Kjeldahl digestion tubes. Ammoniacal nitrogen in the supernatant was determined by the Kjeldahl method (see Section 2.4).

### 2.3. Determination of pH

In the absence of a standard for gastric pH modeling, the AOAC recommended hydrogen ion concentration for gastric drug dissolution testing [11], 0.074 M-HCl with a calculated pH of 1.13 at 0 °C, was used to simulate an optimum gastric medium for peptic digestion. The addition of FDM and protease(s) increased the pH of the digests. Because of meter inaccuracies at low pH, where the measured pH was ≤3.0, the acidity of the gastric medium, prior to inclusion of the FDM and proteases, is reported as a molar concentration (M). A PHM201 pH meter (Radiometer, Copenhagen, Denmark) was used to measure acidity where the pH exceeded 3.0. 

### 2.4. Measurement of Protein Hydrolysis by Kjeldahl

After primary or post-primary digestion, the supernatant was decanted into 250 mL Kjeldahl tubes; the precipitate in the incubation tube was then washed with 10 mL of deionised water, re-centrifuged and that supernatant combined with the initial supernatant. A standard Kjeldahl digestion was followed using steam distillation equipment (UDK126A, VELP Scientifica, Usmate Velate (MB), Italy), trapping the distilled ammonia in boric acid, and titrating with standardised HCl using Tashiro’s indicator.

### 2.5. Measurement of Protein Digestion Efficiency

The fraction of protein hydrolysed expressed as percent ammoniacal N was calculated as:

Fraction of protein hydrolysed = Hydrolysed protein in supernatant

= *Hydrolysed protein in supernatant × 100/ Total protein in FDM, pepsin, and KF*

After a 60 min incubation of 600 mg of FDM with no protease and 20 mL of either 0.074 M HCl, 0.022 M HCl or deionized water, ammoniacal N in the supernatants were 12.95 ± 0.33%; 12.44 ± 0.59% and 12.6 ± 0.67%, respectively. On average, this would comprise about 12.5%, or 64 mg of the 512 mg of protein in 600 mg of FDM in each of the incubations (85.3% protein in FDM). The calculated contribution from pepsin at its standard rate (30 mg at 60.2% protein) would be a further 18 mg, and from KF at its standard rate (100 mg × 4.34% protein) would account for less than an additional 5 mg. Ammoniacal N in the supernatant was typically above 30%, to which pepsin and KF would thus make a minor contribution and were not considered in the calculation.

### 2.6. Sequence of Experiments

A series of five experiments sequentially explored the effects of varying (1) hydrogen ion concentration on the activity of a fixed concentration of KF and no pepsin; (2) KF concentration; (3) gastric incubation time; (4) pepsin concentration at two hydrogen ion concentrations; and (5) pepsin concentration with and without KF on the hydrolysis of FDM.

### 2.7. Statistical Analysis

Unless otherwise stated, data in the text are expressed as means of each treatment ±standard deviation (SD). Paired t tests determined differences between replicates and 95% confidence intervals (CI) of the mean determined. Overall the mean difference between replicates was −0.1418 ± 0.8402 (95% CI −0.3775 to 0.0951, *n* = 49). Independent t tests were used to determine differences between conditions. Analyses were carried out using a combination of IBM^®^ SPSS Statistics version 19.0 (IBM Corp., New York, NY., USA. and Microsoft^®^ Excel^®^ Version 14.2.3 (Microsoft Corporation, Washington DC., USA).

## 3. Results

### 3.1. Effect of Hydrogen Ion Concentration on KF Activity

With 600 mg of FDM, a 60-min incubation in 20 mL of 0.074 M HCl generated 13.56 and 12.94% ammoniacal N in the supernatant, in the presence and absence of 100 mg KF respectively (Table 1). The difference was just significant (*p* = 0.046). Thus KF was essentially inactive under these conditions. However, serial dilution of the HCl almost trebled the ammoniacal N in the supernatant, which reached 37.7% at 0.022 M HCl. The concentration subsequently declined to 27.7% in water alone. A concentration of 0.022 M HCl was chosen as the optimum concentration for KF protease activity. The yield of N in the supernatant from incubating 600 mg FDM in 0.022 M HCl for 60 min with no KF was an expected 12.60% ± 0.69%.

**Table 1:** Effect of acid dilution on protein hydrolysis activity of kiwifruit protease (100 mg KF) on 600 mg of FDM (freeze dried meat) measured by the percentage of ammoniacal nitrogen in the supernatant of the hydrolysates after 60 min incubation at 37°C.

Concentration			Ammoniacal N
HCl:H_2_O	HCl	KF	Mean
(mL:mL)	(M)	(mg)	(%)
20:00	* 0.074	Nil	12.94
20:00	0.074	100	13.56
10:10	0.037	100	19.05
9:11	0.034	100	29.12
8:12	0.030	100	35.62
7:13	0.026	100	36.67
6:14	0.022	100	37.65
5:15	0.019	100	37.04
4:16	0.015	100	32.59
3:17	0.011	100	30.93
2.5:17.5	0.009	100	30.41
1.25:18.75	0.005	100	30.00
00:20	Nil	100	27.68
00:20	Nil	Nil	12.60

The mean difference of all ammoniacal N replicates was 0.15%; 95% CI of difference −0.12 to 0.41; * The AOAC standard *in vitro* gastric molar concentration for drug dissolution testing.

### 3.2. Effect of Changing Concentration of KF on Protein Hydrolysis at Optimal pH

**Figure 1:**
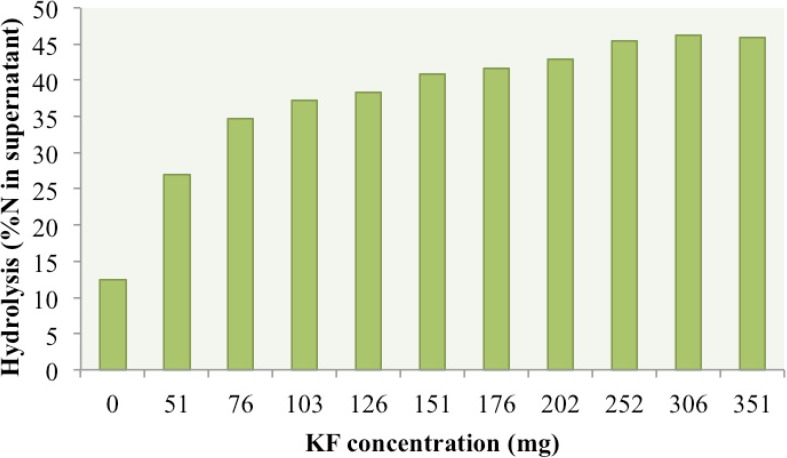
Effect of KF (kiwifruit protease) concentration (mg/digest) on protein hydrolysis of 600 mg FDM (freeze dried meat) measured by percentage of Kjeldahl nitrogen (N) in the supernatant after 60 min incubation in 20 mL 0.022 M HCl at 37 °C.

When the concentration of kiwifruit was sequentially increased from 0 to 350 mg in 20 mL of 0.022 M-HCL (optimal acidity for KF activity) with 600 mg FDM and a 60-min incubation (Figure 1), hydrolysis progressively increased with the greatest rate of increase up to 100 mg KF and saturation occurring when more than 252 mg KF was added (Figure 1). The standard KF concentration in subsequent experiments was set at 100 mg per 600 mg FDM and 20 mL of HCl or water.

### 3.3. Effect of Changing Incubation Time on Protein Hydrolysis

**Figure 2:**
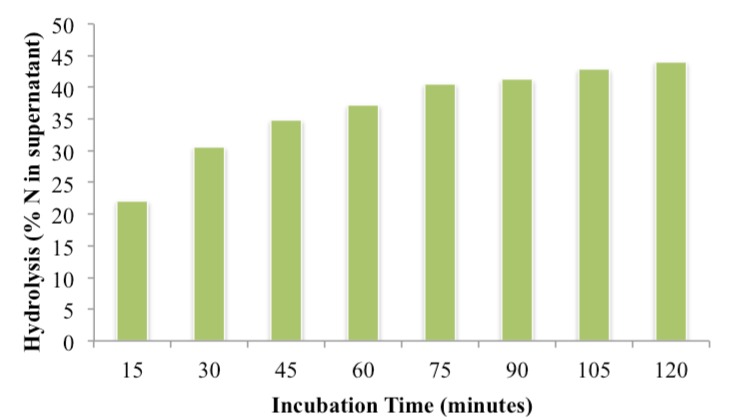
Effect of incubation-time on hydrolysis of FDM (freeze dried meat) by KF (kiwifruit protease) measured by the percentage of ammoniacal nitrogen in the supernatant of the hydrolysate. The reaction mixtures contained 600 mg FDM, 20 mL of 0.022M HCl and 100 mg KF.

Increasing the primary incubation time in 0.022 M HCL with 100 mg of KF but without pepsin increased supernatant ammoniacal N from 37.2% at 60-min to 44.1% at 120 min (Figure 2). Extending digestion for a further 60-min caused a negligible increase to 45.1%. However, extending the primary digestion for the same components in 0.074 M-HCl, the accepted gastric pH (AOAC recommendation), from 60 to 180-min increased N in the supernatant from 13.6% to 30%, this however could have been due to acid-catalysed hydrolysis with time rather than the effect of KF hydrolysis.

Paired sample T-test Mean of difference = 0.124 ± 0.930; 95% CI of the difference −0.657 to 0.905

### 3.4. Effect of Changing Pepsin Concentration on Protein Hydrolysis at Two Hydrogen Ion Concentrations

**Figure 3:**
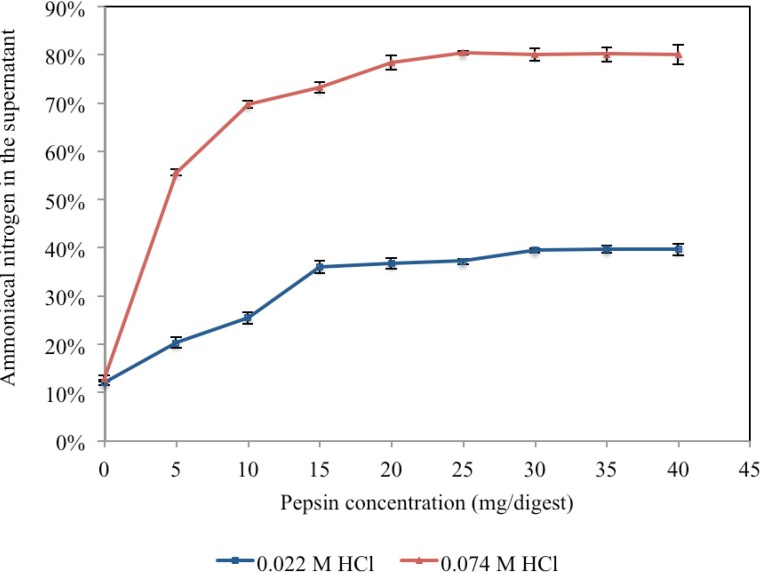
Effect of pepsin concentration on protein hydrolysis of 600 mg freeze-dried meat at two acid concentrations (0.022 M and 0.074 M HCl) measured by percentage of ammoniacal nitrogen in the supernatant of the hydrolysate after 60 min incubation at 37°C. Points on the graph are means of duplicate digests and error are standard deviations

The concentration of pepsin was sequentially increased from 5 to 40 mg in 20 mL of 0.074 M HCl, or 0.022 M HCL (optimal acidity for KF activity) with 600mg FDM and a 60-minute incubation (Figure 3). In 0.074 M HCl with 25 mg of pepsin, protein hydrolysis peaked at 80%. Further increases in pepsin concentration in two repeat experiments gave an average 80.16% ± 0.16% N in the supernatant for 25, 30, 35 and 40 mg pepsin. However, in 0.022 M HCl and more than 15 mg pepsin protein hydrolysis plateaued at 39%. Increasing the pepsin concentration had minimal effect, as the average over the range 25, 30, 35 and 40 mg of pepsin per 600 mg FDM was 39.49% ± 0.43%. A combination of low pepsin concentration (5 mg per 600 mg FDM) and low added acid concentration (0.022 M HCl) resulted in protein hydrolysis of 20.74% ± 0.70%.

### 3.5. Combined Effect of Pepsin and KF on Primary Hydrolysis of Protein.

**Figure 4:**
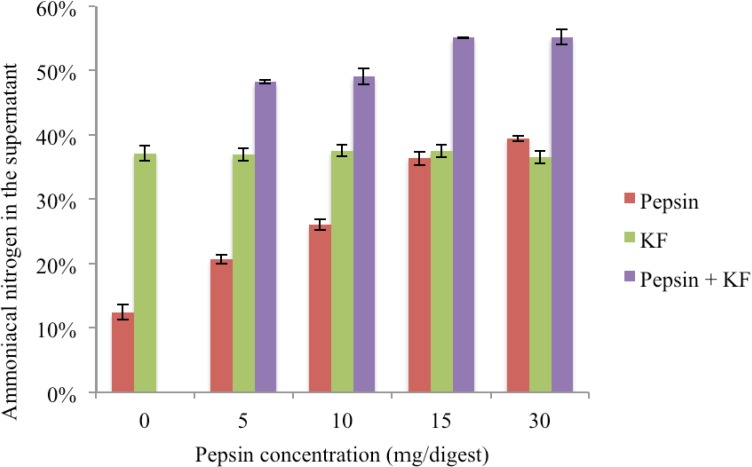
Effect on the hydrolysis of 600 mg of freeze-dried meat (FDM) after 60 min primary digestion with pepsin alone (red bars), or in combination with 100 mg of KF (purple bars). The green bars indicate the hydrolysis effect in 0.022 M HCl of 100 mg of KF on 600 mg of FDM in the absence of pepsin. Incubations each contained 20 mL 0.022 M HCl, 600 mg of FDM, plus various combinations of pepsin ranging from 0 to 30 mg/digest alone or in combination with 100 mg KF. Bars are means of duplicates and errors are standard deviations.

At the optimal pH for pepsin activity (from 0.074 M-HCl), 25 mg of pepsin and 600 mg of FDM, plus 100 mg of KF, resulted in about 80% hydrolysis (data not shown) similar to the effect of pepsin alone (Figure 3). In this high-acid environment, KF had little effect on hydrolysis. However, in the less acidic environment (0.022 M HCl), a combination of 5 mg pepsin and 100 mg of KF resulted in markedly increased hydrolysis. Thus, pepsin alone gave 20.7% ± 0.7% hydrolysis, and the further addition of 100 mg KF increased the hydrolysis to 48.2 ± 0.3% (Figure 4). At higher pepsin concentrations, 10, 15 and 30 mg/incubation in 20 mL of 0.022 M HCl, the addition of 100 mg of KF resulted in similar enhanced hydrolysis, up to 55.2% ± 1.2% of nitrogen in the supernatant with 30 mg of pepsin. Importantly, the greatest increase above pepsin alone was with 5 mg pepsin and 100 mg of KF where hydrolysis more than doubled the pepsin-alone value. In this experiment, each of the 0.022 M acid incubations with the FDM and enzyme additions resulted in a final pH of 3.1 ± 0.1 after 60 min. The addition of 600 mg of FDM to the 20 mL of 0.22 M HCl was the major factor influencing the change in pH from the theoretical 1.65 pH (−logH^+^ conc) of the acid alone to the measured 3.1 pH of the digest after 60 min incubation, whereas the addition of pepsin (5–30 mg) or KF (100 mg) had little effect on the pH of the digest.

## 4. Discussion

This *in vitro* study has shown that in elevated gastric pH conditions, such as may present with compromised gastric function, the addition of kiwifruit protease more than doubled the rate of hydrolysis of meat protein. This novel finding supports the hypothesis that consumption of kiwifruit with a meal can increase the rate and extent of the hydrolysis of protein.

Evidence is also provided that when the added acid concentration decreased from 0.074 M to 0.022 M (HCl), pepsin activity reduced by 50% and kiwifruit activity increased by 300%.

This evidence indicates that kiwifruit may exert its influence on digestion by increasing the extent of protein digestion in the gastric medium when the secretion of pepsin and/or hydrochloric acid is less than optimal. It is reported [12] in a study of 365 healthy subjects, average fasting gastric pH was 2.16 ± 0.11 for men and 2.79 ± 0.18 for women, but in another study of 79 healthy elderly people [13], 11% of the participants recorded fasting gastric pH > 5.0 throughout the study.

Another *in vitro* investigation of kiwifruit activity [14] has reported that when pH of the digestate was maintained at 1.9 with the aid of an automated acid pump, the digestion of beef muscle protein and protein from other sources, was enhanced. The magnitude of the total effect on protein hydrolysis is not reported, however the digestion of Troponin T was increased by 15%.

The present study extends these finding across a range of pH, protease concentrations and incubation times and demonstrates kiwifruit protease achieves considerably greater activity at pH 3.1 compared with pH 1.9. An extensive literature search has found no other reports of kiwifruit activity under varying gastric pH conditions.

Accurately measuring gastric acidity and pepsin concentration *in vivo* without inflicting patient distress, has limited the research on the efficiency of individual’s gastric protein digestion [15]. As a result there is no accepted standard for pH or enzyme concentrations for *in vitro* gastric studies. The AOAC 1995 standard for testing drug dissolution in an *in vitro* gastric medium [11], specifies 0.0744 M HCl, which has a pH value of 1.13 before protein addition. Other researchers [16] have used similar acid concentration for protein digestion, as used in this present study. Takumi *et al.* (2000), selected 0.1 M HCl (pH 1.0) for a pH-controlled study but emphasised the need to establish standard guidelines for *in vitro* gastric pH research [17]. 

The study demonstrated that kiwifruit had little effect on post-primary digestion of meat protein hydrolysis where pH was 6.4, indicating the protease effect of kiwifruit on protein hydrolysis is more likely to occur in the stomach rather than the small intestine or the colon where the normal pH range is 6.4–7.0. The activity decline observed, as pH increased above 3.6 supports the hypothesis that elevated gastric pH and pepsin insufficiency could be determining factors in kiwifruit’s effect on digestion. The increase in protein digestion efficiency, observed when KF concentration was increased, may indicate *in vivo*, where consumption of one kiwifruit fails to relieve digestive discomfort, increasing the dose might improve the outcome.

If these *in vitro* results are replicated *in vivo*, this study indicates that kiwifruit is likely to exert its influence on digestion by increasing the extent of protein digestion in the gastric medium when the ratio of pepsin to substrate is less than optimal, and gastric pH is elevated to the extent that it compromises the activity of pepsin, but not to the extent that it inactivates the kiwifruit protease.

There are a number of strengths and limitations in the study. One strength is that the conditions of the experiments are all within pathophysiological ranges such as might be found with functional GI disorder. The standard concentration of KF used in these experiments was 100 mg KF: 600 mg FDM. This is equivalent to 1250 mg of fresh kiwifruit to 2 g of meat. A medium sized kiwifruit will yield approximately 60g of flesh, which would provide the same KF concentration used in these experiments, for 96 g of meat. Assuming meat is 25%–30% protein and the daily requirement is 1 g of protein/kg of body weight then one kiwifruit would provide treatment for 40% of the daily protein requirements of a 75 kg person based on the dose used in this study.

Selecting an appropriate baseline pH for *in vitro* studies of gastric digestion can be contentious as studies show that gastric pH varies significantly between individuals of similar age but more commonly between young and elderly people [13]. The starting point for this study was 0.074 M HCl and while a dynamic model to maintain gastric pH throughout the digestion was not used, restricting experiments to one acid concentration would have masked one of the more important findings of the study. The optimal acid concentration for KF activity was 0.022 M HCl (pH 1.66), before addition of FDM and protease, which together increased the pH to 3.1 after their addition.

We do not know the specific rate of human pepsin production but it is estimated that a healthy adult secretes about 20,000 to 30,000 units of pepsin activity per day, which is thought to be about 10 mg equivalent of commercially available pepsin used for this *in vitro* study [18]. If an adult consumes 75 g of protein daily then the ratio of protein to pepsin is about 3 mg protein/unit of activity, or 10 g of protein: 0.18 mg of commercial pepsin. Our experiments used the equivalent of 1 g of protein to 10–60 mg of pepsin. The use of higher concentrations of enzyme to substrate for *in vitro* experiments is not uncommon. Porcine pepsin, as used in this study, has been observed to display greater activity than human pepsin [19]. As a result, *in vivo* hydrolysis by kiwifruit may vary from these *in vitro* results.

These findings do not indicate why kiwifruit provides relief from the discomfort of functional gastrointestinal disorder but the following speculation attempts to provide a rationale for further testing of the hypothesis that kiwifruit provides relief of the symptoms of functional GI disorder.

Primary protein digestion efficiency *in vitro* was determined by pepsin concentration and the acidity of the gastric milieu. Chronic gastritis, a condition not uncommon among the elderly [20], can result in hypochlorhydria (gastric acid insufficiency), while achlorhydria (an absence of gastric acid) represents the extreme outcome of gastritis. Even small variations in gastric pH and pepsin concentration in the present *in vitro* study had a marked influence on protein digestion efficiency and as acid and pepsin concentration were decreased, peptic protein hydrolysis became progressively less efficient. This is in agreement with a study on pH effects on pepsin activity conducted by Al-Janabi *et al.* (1972) that demonstrated pepsin activity on protein hydrolysis was 100 times faster at pH 1.0 than at pH 4.6 [21]. If the pH effect is replicated *in vivo*, this finding may have implications for some age associated diseases such as cancer, sarcopenia and reduced immune function [22,23].

The prevalence of elevated gastric pH is thought to be high in elderly people as a result of pathology and medication rather than physiology [24]. Constipation, a symptom of functional GI disorder, is also more prevalent amongst the elderly, possibly for the same reasons [25]. Whilst constipation, as an outcome of inefficient protein digestion, is not identified by Jahan-Mihan *et al.* [26] these researchers postulate that within the GI tract, peptide products of protein hydrolysis affect several regulatory functions. The mechanism they propose is that the peptide products stimulate receptors to initiate the secretion of hormones that affect gastric emptying and GI transport and absorption mechanisms as well as transmitting neural signals to the brain. The peptide products also have a modifying effect on the micro flora. These effects, combined with the finding that kiwifruit increases protein hydrolysis constrained by acidity and pepsin inadequacy (this study), and that kiwifruit relieves constipation [27,28], lends support to the hypothesis that kiwifruit relieves constipation by increasing gastric protein hydrolysis.

The aim of treatment with proton pump inhibiting (PPI) medication is to maintain gastric acidity above pH 4 [29]. A concern expressed by a number of researchers [30,31] is that PPI treatment limits gastric hydrolysis of protein. This increases the loading on pancreatic and brush border protease production. Failure of these secondary sources to compensate for inefficient primary gastric hydrolysis may have implications such as infectious complications and nutritional deficiencies. This is an area of proposed future research.

Due in part to the invasive nature of current testing methods, regular measurement of individual’s fasting gastric pH and re-acidification efficiency are not currently a feature of routine health checks, making it conceivable that a pernicious deterioration in protein digestion efficiency over time could go undetected. If sarcopenia in the elderly results from a deterioration of protein assimilation rather than a dietary protein deficiency, this raises the possibility of inefficient peptic protein digestion as a contributing factor in the onset of sarcopenia, and possibly other age associated conditions.

Extrapolations from the findings of this study and the literature reviewed, indicate that relief from functional GI disorder, associated with kiwifruit consumption, may be a result of three factors alone or in combination: first, improved gastric protein hydrolysis efficiency when gastric acidity is elevated; second, a reduction in the quantum of undigested protein entering the duodenum reducing reliance on pancreatic digestion to compensate for impaired gastric digestion; third, reduced protein in the chyme entering the colon, resulting in beneficial, compositional changes in gut micro-flora populations.

## 5. Conclusions

The consumption of kiwifruit with a meal may increase the rate of hydrolysis of protein, particularly if gastric pH is abnormally elevated, which may explain how kiwifruit relieves functional GI disorder. Further *in vivo* investigations of gastric digestion efficiency are warranted.

## References

[1] Malagelada C., Malagelada J.-R. (2010). The dysfunctional gut. Curr. Gastroenterol. Rep..

[2] Miszputen S.J. (2008). Constipation in women. Rev. Bras. Med..

[3] D’Souza A.L. (2007). Ageing and the gut. Postgrad. Med. J..

[4] Tiihonen K., Tynkkynen S., Ouwehand A., Ahlroos T., Rautonen N. (2008). The effect of ageing with and without non-steroidal anti-inflammatory drugs on gastrointestinal microbiology and immunology. Br. J. Nutr..

[5] Wyeth J.W., O'Rourke M., Wong J.S., Keating J.P. (2000).

[6] Rush E.C., Patel M., Plank L.D., Ferguson L.R. (2002). Kiwifruit promotes laxation in the elderly. Asia Pac. J. Clin. Nutr..

[7] Uebaba K., Urata T., Suzuki N., Arai T., Strong J.M., Oono S., Hayashi H. (2009). Mild Laxative and QOL-improving Effects of Kiwi Fruit Extract in the Elderly—An Explanatory Study on Effectiveness and Safety. Jpn. J. Compl. Altern. Med..

[8] Wyeth J.W. (2011). Functional gastrointestinal disorders in New Zealand. J. Gastroenterol. Hepatol..

[9] Lewis D.A., Luh B.S. (1987). Development and distribution of actinidin in kiwifruit (*Actinidia chinensis*) and its partial characterization. J. Food Biochem..

[10] (2011). Fox Scientific. Kjeldahl Standard Operating Procedure.

[11] AOAC International (1995). Official Methods of Analysis of AOAC International.

[12] Feldman M., Barnett C. (1991). Fasting gastric pH and its relationship to true hypochlorhydria in humans. Dis. Sci..

[13] Clarkston W.K., Pantano M.M., Morley J.E., Horowitz M., Littlefield J.M., Burton F.R. (1997). Evidence for the anorexia of aging: Gastrointestinal transit and hunger in healthy elderly *vs.* young adults. Am. J. Physiol..

[14] Kaur L., Rutherfurd S.M., Moughan P.J., Drummond L.N., Boland M.J. (2010). Actinidin enhances gastric protein digestion as assessed using an *in vitro* gastric digestion model. J. Agric. Food Chem..

[15] Goetze O., Treier R., Fox M., Steingoetter A., Fried S.M., Boesiger P., Schwizer W. (2009). The effect of gastric secretion on gastric physiology and emptying in the fasted and fed state assessed by magnetic resonance imaging. Neurogastroenterol. Motil..

[16] Bellaver C., Zanotto D.L., Guidoni A.L., Klein C.H. (2000). *In vitro* solubility of meat and bone meal protein with different pepsin concentrations. Cienc. Rural..

[17] Takumi K., de Jonge R., Havelaar A. (2000). Modelling inactivation of Escherichia coli by low pH: Application to passage through the stomach of young and elderly people. J. Appl. Microbiol..

[18] Wickham M., Faulks R., Mills C. (2009). *In vitro* digestion methods for assessing the effect of food structure on allergen breakdown. Mol. Nutr. Food Res..

[19] Eriksen E.K., Holm H., Jensen E., Aaboe R., Devold T.G., Jacobsen M., Vegarud G.E. (2010). Different digestion of caprine whey proteins by human and porcine gastrointestinal enzymes. Br. J. Nutr..

[20] Adamu M.A., Weck M.N., Rothenbacher D., Brenner H. (2011). Incidence and risk factors for the development of chronic atrophic gastritis: Five year follow-up of a population-based cohort study. Int. J. Cancer.

[21] Al-Janabi J., Hartsuck J.A., Tang J. (1972). Kinetics and mechanism of pepsinogen activation. J. Biol. Chem..

[22] Derakhshan M.H., Malekzadeh R., Watabe H., Yazdanbod A., Fyfe V., Kazemi A., Rakhshani N., Didevar R., Sotoudeh M., Zolfeghari A.A. (2008). Combination of gastric atrophy, reflux symptoms and histological subtype indicates two distinct aetiologies of gastric cardia cancer. Gut.

[23] Ito T., Jensen R.T. (2010). Association of long-term proton pump inhibitor therapy with bone fractures and effects on absorption of calcium, vitamin B12, iron, and magnesium. Curr. Gastroenterol. Rep..

[24] Salles N. (2009). Is stomach spontaneously ageing? Pathophysiology of the ageing stomach. Best Pract. Res. Clin. Gastroenterol..

[25] Schaefer D.C., Cheskin L.J. (1998). Constipation in the elderly. Am. Fam. Physician.

[26] Jahan-Mihan A., Luhovyy B.L., El Khoury D., Anderson G.H. (2011). Dietary proteins as determinants of metabolic and physiologic functions of the gastrointestinal tract. Nutrients.

[27] Chan A.O.O., Leung G., Tong T., Wong N.Y.H. (2007). Increasing dietary fiber intake in terms of kiwifruit improves constipation in Chinese patients. World J. Gastroenterol..

[28] Chang C.-C., Lin Y.-T., Lu Y.-T., Liu Y.-S., Liu J.-F. (2010). Kiwifruit improves bowel function in patients with irritable bowel syndrome with constipation. Asia Pac. J. Clin. Nutr..

[29] Shi S., Klotz U. (2008). Proton pump inhibitors: An update of their clinical use and pharmacokinetics. Eur. J. Clin. Pharmacol..

[30] Raghunath A.S., O’Morain C., McLough R.C. (2005). Review article: The long-term use of proton-pump inhibitors. Aliment. Pharmacol. Therapeutics.

[31] Ali T., Roberts D.N., Tierney W.M. (2009). Long-term safety concerns with proton pump inhibitors. Am. J. Med..

